# 4-(4,4-Difluoro-1,3,5,7-tetra­methyl-3a-aza-4a-azonia-4-borata-*s*-indacen-8-yl)benzonitrile

**DOI:** 10.1107/S1600536811009457

**Published:** 2011-03-19

**Authors:** Yuting Chen, Jianzhuang Jiang

**Affiliations:** a Department of Chemistry, Shandong University, Jinan 250100, People’s Republic of China; b Department of Chemistry, Dezhou University, Dezhou 253023, People’s Republic of China

## Abstract

The title compound, C_20_H_18_BF_2_N_3_, contains one C_9_BN_2_ (Bodipy) framework and one cyano­benzyl group. The Bodipy framework is essentially planar with a maximum deviation of 0.041 (2) Å. The introduction of two methyl groups at positions 1 and 7 of *s*-indacene in the Bodipy unit results in almost orthogonal configuration between the mean plane of the Bodipy unit and the cyano­benzyl group [dihedral angle = 89.78 (4)°].

## Related literature

For the structures and optical properties of Bodipy dyes, see: Loudet & Burgess (2007 ▶) and Feng *et al.* (2008 ▶), respectively. For the relation between the crystal structures and optical properties of Bodipy compounds, see: Cui *et al.* (2007 ▶); Broring *et al.*(2008 ▶).
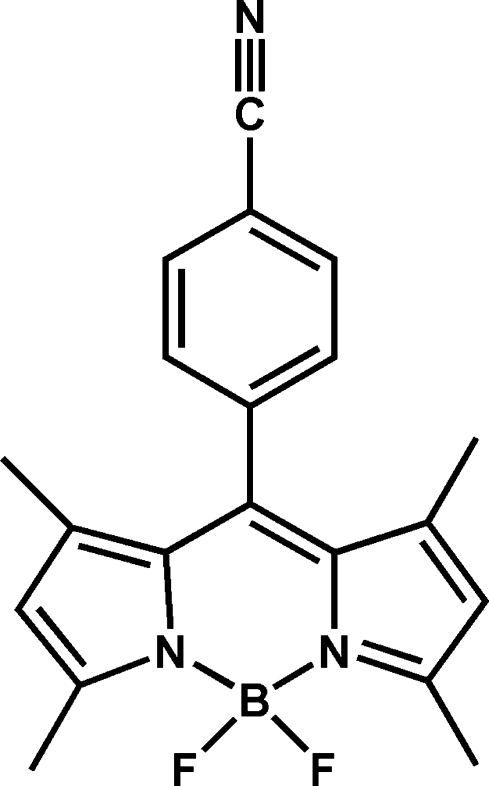


## Experimental

### 

#### Crystal data

C_20_H_18_BF_2_N_3_
*M* *_r_* = 349.18
Monoclinic, 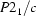

*a* = 7.6498 (3) Å
*b* = 11.3715 (5) Å
*c* = 21.555 (1) Å
β = 92.008 (4)°
*V* = 1873.91 (14) Å^3^
*Z* = 4
Mo *K*α radiation
μ = 0.09 mm^−1^
*T* = 293 K
0.20 × 0.18 × 0.16 mm

#### Data collection

Bruker SMART 1000 CCD area-detector diffractometer
Absorption correction: multi-scan (*SADABS*; Sheldrick, 1996 ▶) *T*_min_ = 0.983, *T*_max_ = 0.986
7304 measured reflections
3286 independent reflections
2240 reflections with *I* > 2σ(*I*)
*R*_int_ = 0.028

#### Refinement

*R*[*F* ^2^ > 2σ(*F* ^2^)] = 0.065
*wR*(*F* ^2^) = 0.195
*S* = 1.07
3286 reflections
235 parameters
H-atom parameters constrained
Δρ_max_ = 0.30 e Å^−3^
Δρ_min_ = −0.25 e Å^−3^

### 

Data collection: *SMART* (Bruker, 1996 ▶); cell refinement: *SAINT* (Bruker, 1996 ▶); data reduction: *SAINT*; program(s) used to solve structure: *SHELXS97* (Sheldrick, 2008 ▶); program(s) used to refine structure: *SHELXL97* (Sheldrick, 2008 ▶); molecular graphics: *SHELXTL* (Sheldrick, 2008 ▶); software used to prepare material for publication: *SHELXTL*.

## Supplementary Material

Crystal structure: contains datablocks I, global. DOI: 10.1107/S1600536811009457/aa2003sup1.cif

Structure factors: contains datablocks I. DOI: 10.1107/S1600536811009457/aa2003Isup2.hkl

Additional supplementary materials:  crystallographic information; 3D view; checkCIF report

## Figures and Tables

**Table 1:** Selected bond lengths (Å)

F2—B1	1.382 (4)
N1—C5	1.350 (3)
N1—C9	1.400 (3)
N1—B1	1.535 (4)
F1—B1	1.384 (3)
N2—C3	1.346 (3)
N2—C10	1.408 (3)
N2—B1	1.545 (4)
C8—C10	1.390 (3)
C8—C9	1.398 (3)
C10—C1	1.429 (4)
C9—C7	1.426 (4)

**Fig. 1.**
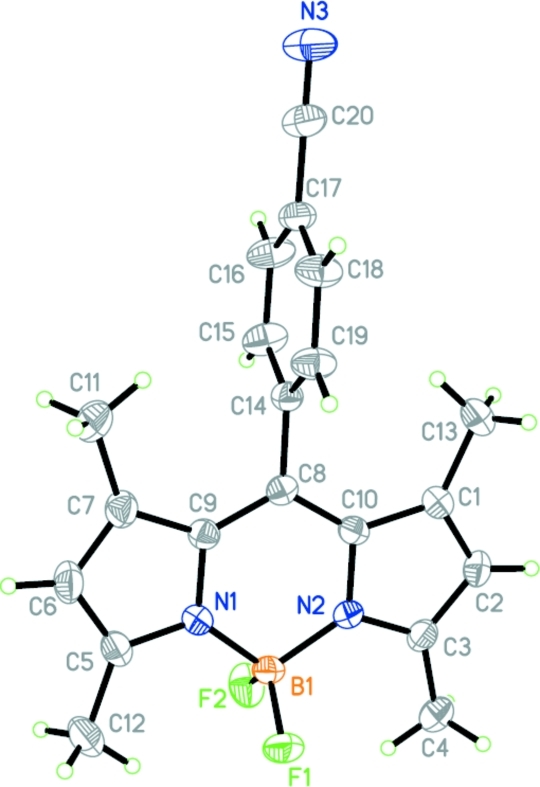
The molecular structure of the title compound. Displacement ellipsoids are drawn at 30% probability level.

## supplementary crystallographic information

### Comment

Fluorescent dyes, especially 4,4-difluoro-4-bora-3a,4a-diaza-s-indacene (Bodipy), have been extensively studied due to their advantageous photospectral properties including high photostability, sharp absorption and emission bands, relatively high absorption coefficient, high fluorescence quantum yields, and the extraordinary feature of excitation/emission wavelengths in the visible region (Loudet *et al.*, 2007; Feng *et al.* 2008). Single crystal structural studies of these compounds have become increasingly important in revealing precisely the relation between their optical properties and molecular structures (Cui *et al.*, 2007). As an extension of our work on the structural characterization of this field, the title compound, (I), is synthesized and characterized by *x*-ray diffraction, as shown in Fig. 1.

The compound (I) crystallizes in the monoclinic system with only one molecule per unit cell, and contains mainly one C_9_BN_2_ (Bodipy) framework and one cyanobenzyl unit. The C_9_BN_2_ (Bodipy) framework consisting of one central six-membered and two adjacent five-membered rings is essentially flat, with the maximum deviation from the least-squares mean plane being 0.041 Å. As shown in Table 1, the C—C and C—N bond lengths within C_9_BN_2_ (Bodipy) framework, without any clear distinction between single and double bonds, indicate the strongly delocalized π-system nature of the Bodipy framework. However, this π-electron delocalization is interrupted between the two B—N bonds (Broring *et al.* 2008). More interestingly, the introduction of two methyl groups onto C-1 and C-7 atoms in Bodipy moiety results in almostly orthogonal configuration between the mean plane of Bodipy moiety and cyanobenzyl unit with the dihedral angle of 89.78 (4)°, indicating the almost non-electronic coupling nature between these moieties. (Loudet *et al.*, 2007).

### Experimental

To the mixture of 4-cyanobenzaldehyde (131 mg, 1 mmol) and 2,4-dimethylpyrrole (190 mg, 2.00 mmol) dissolved in CH_2_Cl_2_ (100 ml), one drop of TFAwas added. The resulting mixture was then stirred at room temperature under N_2_ atmosphere. When thin-layer chromatography (TLC) monitoring indicated the complete consumption of the aldehyde, a solution of DDQ (227 mg, 1 mmol) in CH_2_Cl_2_ (40 ml) was added and the reaction mixture was further stirred for another 15 min. After the addition of N, *N*-diisopropylethylamine (DIEA) (2 ml) into the mixture for 15 min, the BF_3_—OEt_2_ (2.0 ml) was added into the reaction mixture and stirring was continued for another 30 min. The resulting mixture was evaporated, and the residue was chromatographed on a silica gel column using CH_2_Cl_2_/hexane (1:1) as eluent. Repeated chromatography followed by recrystallization from CH_2_Cl_2_ and hexane gave the target compound as black-red crystals. Yield: 104 mg, 15%. Anal. for C_20_H_18_BF_2_N_3_: Calc. C, 68.79; H, 5.20; N, 12.03; Found: C, 68.10; H, 5.17; N, 12.81%. The No. of CCDC: 814146.

### Refinement

All H atoms were placed in geometrically idealized positions and treated as riding on their parent atoms with C–H distances of 0.96Å and *U*_iso_(H)=1.5*U*_eq_(C) for methyl H-atoms and C–H distances of 0.93Å and *U*_iso_(H)=1.2*U*_eq_(C) for other H-atoms.

### Crystal data

**Table:**

C_20_H_18_BF_2_N_3_	*F*(000) = 728
*M**_r_* = 349.18	*D*_x_ = 1.238 Mg m^−^^3^
Monoclinic, *P*2_1_/*c*	Mo *K*α radiation, λ = 0.71073 Å
Hall symbol: -P 2ybc	Cell parameters from 3286 reflections
*a* = 7.6498 (3) Å	θ = 2.3–25.0°
*b* = 11.3715 (5) Å	µ = 0.09 mm^−^^1^
*c* = 21.555 (1) Å	*T* = 293 K
β = 92.008 (4)°	Block, red
*V* = 1873.91 (14) Å^3^	0.20 × 0.18 × 0.16 mm
*Z* = 4	

### Data collection

**Table:**

Bruker SMART 1000 CCD area-detector diffractometer	3286 independent reflections
Radiation source: fine-focus sealed tube	2240 reflections with *I* > 2σ(*I*)
graphite	*R*_int_ = 0.028
φ and ω scans	θ_max_ = 25.0°, θ_min_ = 3.2°
Absorption correction: multi-scan (*SADABS*; Sheldrick, 1996)	*h* = −9→8
*T*_min_ = 0.983, *T*_max_ = 0.986	*k* = −13→13
7304 measured reflections	*l* = −25→16

### Refinement

**Table:**

Refinement on *F*^2^	Primary atom site location: structure-invariant direct methods
Least-squares matrix: full	Secondary atom site location: difference Fourier map
*R*[*F*^2^ > 2σ(*F*^2^)] = 0.065	Hydrogen site location: inferred from neighbouring sites
*wR*(*F*^2^) = 0.195	H-atom parameters constrained
*S* = 1.07	*w* = 1/[σ^2^(*F*_o_^2^) + (0.1059*P*)^2^ + 0.276*P*] where *P* = (*F*_o_^2^ + 2*F*_c_^2^)/3
3286 reflections	(Δ/σ)_max_ < 0.001
235 parameters	Δρ_max_ = 0.30 e Å^−^^3^
0 restraints	Δρ_min_ = −0.25 e Å^−^^3^

### Special details

**Table:**

Geometry. All s.u.'s (except the s.u. in the dihedral angle between two l.s. planes) are estimated using the full covariance matrix. The cell s.u.'s are taken into account individually in the estimation of s.u.'s in distances, angles and torsion angles; correlations between s.u.'s in cell parameters are only used when they are defined by crystal symmetry. An approximate (isotropic) treatment of cell s.u.'s is used for estimating s.u.'s involving l.s. planes.
Refinement. Refinement of *F*^2^ against ALL reflections. The weighted *R*-factor *wR* and goodness of fit *S* are based on *F*^2^, conventional *R*-factors *R* are based on *F*, with *F* set to zero for negative *F*^2^. The threshold expression of *F*^2^ > σ(*F*^2^) is used only for calculating *R*-factors(gt) *etc*. and is not relevant to the choice of reflections for refinement. *R*-factors based on *F*^2^ are statistically about twice as large as those based on *F*, and *R*- factors based on ALL data will be even larger.

### Fractional atomic coordinates and isotropic or equivalent isotropic displacement parameters (Å^2^)

**Table:**

	*x*	*y*	*z*	*U*_iso_*/*U*_eq_	
F2	0.6736 (2)	1.18071 (15)	0.82647 (9)	0.0706 (6)	
N1	0.7514 (3)	0.97876 (19)	0.81048 (9)	0.0409 (6)	
F1	0.8040 (3)	1.13260 (16)	0.73783 (8)	0.0750 (6)	
N2	0.9776 (3)	1.13074 (18)	0.83306 (9)	0.0384 (5)	
C17	1.2845 (4)	0.6972 (2)	0.99641 (13)	0.0491 (7)	
C8	1.0075 (3)	0.9342 (2)	0.87582 (11)	0.0376 (6)	
C14	1.1043 (3)	0.8502 (2)	0.91690 (12)	0.0391 (6)	
C10	1.0737 (3)	1.0468 (2)	0.86812 (11)	0.0388 (6)	
C9	0.8499 (3)	0.8989 (2)	0.84668 (11)	0.0405 (6)	
C20	1.3795 (5)	0.6205 (3)	1.03874 (14)	0.0619 (9)	
C18	1.1552 (4)	0.7687 (3)	1.01831 (13)	0.0623 (9)	
H18	1.1293	0.7667	1.0601	0.075*	
C16	1.3223 (4)	0.7006 (3)	0.93441 (14)	0.0593 (8)	
H16	1.4078	0.6513	0.9191	0.071*	
C15	1.2329 (4)	0.7774 (3)	0.89494 (13)	0.0555 (8)	
H15	1.2595	0.7801	0.8532	0.067*	
C2	1.2232 (4)	1.2167 (3)	0.86865 (14)	0.0548 (8)	
H2	1.3084	1.2739	0.8758	0.066*	
C5	0.6123 (3)	0.9205 (3)	0.78539 (13)	0.0495 (7)	
C19	1.0646 (4)	0.8426 (3)	0.97863 (13)	0.0579 (8)	
H19	0.9748	0.8885	0.9936	0.069*	
C4	0.9998 (4)	1.3421 (3)	0.80490 (16)	0.0627 (9)	
H4A	0.8893	1.3266	0.7840	0.094*	
H4B	1.0814	1.3701	0.7754	0.094*	
H4C	0.9849	1.4008	0.8363	0.094*	
C1	1.2306 (3)	1.1032 (3)	0.89043 (13)	0.0484 (7)	
C7	0.7648 (4)	0.7874 (2)	0.84295 (14)	0.0510 (7)	
C6	0.6190 (4)	0.8041 (3)	0.80502 (15)	0.0588 (8)	
H3	0.5373	0.7465	0.7942	0.071*	
C3	1.0675 (4)	1.2325 (2)	0.83408 (12)	0.0461 (7)	
B1	0.7971 (4)	1.1087 (3)	0.80066 (14)	0.0441 (8)	
C11	0.8200 (5)	0.6725 (3)	0.87155 (19)	0.0788 (11)	
H5A	0.9251	0.6836	0.8965	0.118*	
H5B	0.8411	0.6164	0.8393	0.118*	
H5C	0.7290	0.6438	0.8971	0.118*	
C13	1.3786 (4)	1.0532 (3)	0.92990 (17)	0.0722 (10)	
H13A	1.3544	0.9724	0.9392	0.108*	
H13B	1.3907	1.0971	0.9679	0.108*	
H13C	1.4852	1.0582	0.9078	0.108*	
C12	0.4752 (4)	0.9784 (3)	0.74490 (17)	0.0751 (10)	
H1A	0.5045	1.0598	0.7395	0.113*	
H1B	0.3640	0.9725	0.7640	0.113*	
H1C	0.4690	0.9402	0.7052	0.113*	
N3	1.4528 (4)	0.5619 (3)	1.07363 (14)	0.0836 (10)	

### Atomic displacement parameters (Å^2^)

**Table:**

	*U*^11^	*U*^22^	*U*^33^	*U*^12^	*U*^13^	*U*^23^
F2	0.0473 (9)	0.0506 (10)	0.1138 (16)	0.0101 (8)	0.0003 (10)	−0.0057 (10)
N1	0.0394 (11)	0.0438 (13)	0.0393 (12)	−0.0006 (10)	−0.0026 (9)	0.0010 (10)
F1	0.0938 (14)	0.0822 (13)	0.0473 (11)	−0.0220 (11)	−0.0203 (9)	0.0224 (9)
N2	0.0407 (11)	0.0357 (12)	0.0388 (12)	0.0002 (9)	0.0007 (9)	0.0048 (10)
C17	0.0574 (17)	0.0432 (16)	0.0460 (17)	0.0028 (13)	−0.0073 (13)	0.0088 (13)
C8	0.0411 (13)	0.0378 (15)	0.0342 (13)	0.0026 (11)	0.0065 (11)	−0.0002 (11)
C14	0.0407 (13)	0.0374 (14)	0.0390 (15)	0.0019 (11)	−0.0008 (11)	0.0004 (12)
C10	0.0366 (12)	0.0439 (15)	0.0358 (14)	0.0035 (11)	0.0004 (10)	0.0011 (12)
C9	0.0402 (13)	0.0401 (15)	0.0412 (14)	0.0019 (11)	0.0003 (11)	0.0005 (12)
C20	0.084 (2)	0.0478 (18)	0.0534 (19)	0.0053 (17)	−0.0091 (17)	0.0070 (16)
C18	0.093 (2)	0.0577 (19)	0.0371 (16)	0.0218 (18)	0.0102 (16)	0.0069 (15)
C16	0.0633 (18)	0.064 (2)	0.0510 (18)	0.0255 (16)	0.0034 (14)	0.0056 (15)
C15	0.0631 (18)	0.065 (2)	0.0383 (15)	0.0189 (16)	0.0066 (13)	0.0074 (14)
C2	0.0499 (16)	0.0527 (19)	0.0617 (19)	−0.0188 (14)	−0.0007 (14)	0.0050 (15)
C5	0.0418 (14)	0.0539 (18)	0.0522 (16)	−0.0050 (13)	−0.0060 (13)	−0.0024 (14)
C19	0.078 (2)	0.0560 (19)	0.0410 (16)	0.0208 (16)	0.0178 (15)	0.0044 (14)
C4	0.068 (2)	0.0479 (18)	0.072 (2)	−0.0081 (16)	0.0016 (17)	0.0148 (16)
C1	0.0425 (14)	0.0533 (18)	0.0489 (16)	−0.0033 (13)	−0.0048 (12)	0.0027 (14)
C7	0.0492 (15)	0.0412 (16)	0.0626 (19)	−0.0040 (13)	−0.0011 (14)	−0.0014 (14)
C6	0.0496 (16)	0.0511 (19)	0.075 (2)	−0.0158 (14)	−0.0031 (15)	−0.0054 (16)
C3	0.0501 (15)	0.0439 (16)	0.0444 (16)	−0.0074 (13)	0.0052 (12)	0.0043 (13)
B1	0.0478 (17)	0.0419 (18)	0.0421 (17)	0.0003 (14)	−0.0056 (14)	0.0069 (15)
C11	0.079 (2)	0.0441 (19)	0.112 (3)	−0.0110 (17)	−0.013 (2)	0.0184 (19)
C13	0.0506 (17)	0.078 (2)	0.086 (2)	−0.0111 (16)	−0.0224 (17)	0.0132 (19)
C12	0.0591 (19)	0.077 (2)	0.086 (2)	−0.0084 (18)	−0.0288 (18)	0.0025 (19)
N3	0.111 (2)	0.0673 (19)	0.0713 (19)	0.0198 (18)	−0.0186 (17)	0.0168 (16)

### Geometric parameters (Å, °)

**Table:**

F2—B1	1.382 (4)	C2—C1	1.374 (4)
N1—C5	1.350 (3)	C2—C3	1.394 (4)
N1—C9	1.400 (3)	C2—H2	0.9300
N1—B1	1.535 (4)	C5—C6	1.390 (4)
F1—B1	1.384 (3)	C5—C12	1.494 (4)
N2—C3	1.346 (3)	C19—H19	0.9300
N2—C10	1.408 (3)	C4—C3	1.482 (4)
N2—B1	1.545 (4)	C4—H4A	0.9600
C17—C18	1.376 (4)	C4—H4B	0.9600
C17—C16	1.378 (4)	C4—H4C	0.9600
C17—C20	1.440 (4)	C1—C13	1.504 (4)
C8—C10	1.390 (3)	C7—C6	1.373 (4)
C8—C9	1.398 (3)	C7—C11	1.500 (4)
C8—C14	1.483 (3)	C6—H3	0.9300
C14—C19	1.378 (4)	C11—H5A	0.9600
C14—C15	1.382 (4)	C11—H5B	0.9600
C10—C1	1.429 (4)	C11—H5C	0.9600
C9—C7	1.426 (4)	C13—H13A	0.9600
C20—N3	1.138 (4)	C13—H13B	0.9600
C18—C19	1.371 (4)	C13—H13C	0.9600
C18—H18	0.9300	C12—H1A	0.9600
C16—C15	1.384 (4)	C12—H1B	0.9600
C16—H16	0.9300	C12—H1C	0.9600
C15—H15	0.9300		
			
C5—N1—C9	107.8 (2)	C3—C4—H4B	109.5
C5—N1—B1	126.7 (2)	H4A—C4—H4B	109.5
C9—N1—B1	125.5 (2)	C3—C4—H4C	109.5
C3—N2—C10	108.5 (2)	H4A—C4—H4C	109.5
C3—N2—B1	126.5 (2)	H4B—C4—H4C	109.5
C10—N2—B1	125.0 (2)	C2—C1—C10	106.4 (2)
C18—C17—C16	119.6 (2)	C2—C1—C13	124.7 (3)
C18—C17—C20	119.5 (3)	C10—C1—C13	128.8 (3)
C16—C17—C20	120.9 (3)	C6—C7—C9	105.7 (2)
C10—C8—C9	121.5 (2)	C6—C7—C11	125.2 (3)
C10—C8—C14	119.2 (2)	C9—C7—C11	129.1 (3)
C9—C8—C14	119.2 (2)	C7—C6—C5	109.5 (2)
C19—C14—C15	118.5 (2)	C7—C6—H3	125.3
C19—C14—C8	119.6 (2)	C5—C6—H3	125.3
C15—C14—C8	121.9 (2)	N2—C3—C2	108.9 (2)
C8—C10—N2	120.2 (2)	N2—C3—C4	123.1 (3)
C8—C10—C1	132.8 (2)	C2—C3—C4	127.9 (3)
N2—C10—C1	107.0 (2)	F2—B1—F1	109.1 (2)
C8—C9—N1	120.2 (2)	F2—B1—N1	110.6 (2)
C8—C9—C7	131.7 (2)	F1—B1—N1	109.9 (2)
N1—C9—C7	108.0 (2)	F2—B1—N2	109.5 (2)
N3—C20—C17	177.9 (4)	F1—B1—N2	110.3 (2)
C19—C18—C17	120.1 (3)	N1—B1—N2	107.3 (2)
C19—C18—H18	119.9	C7—C11—H5A	109.5
C17—C18—H18	119.9	C7—C11—H5B	109.5
C17—C16—C15	119.9 (3)	H5A—C11—H5B	109.5
C17—C16—H16	120.1	C7—C11—H5C	109.5
C15—C16—H16	120.1	H5A—C11—H5C	109.5
C14—C15—C16	120.7 (3)	H5B—C11—H5C	109.5
C14—C15—H15	119.7	C1—C13—H13A	109.5
C16—C15—H15	119.7	C1—C13—H13B	109.5
C1—C2—C3	109.1 (2)	H13A—C13—H13B	109.5
C1—C2—H2	125.4	C1—C13—H13C	109.5
C3—C2—H2	125.4	H13A—C13—H13C	109.5
N1—C5—C6	109.0 (2)	H13B—C13—H13C	109.5
N1—C5—C12	123.0 (3)	C5—C12—H1A	109.5
C6—C5—C12	128.0 (3)	C5—C12—H1B	109.5
C18—C19—C14	121.1 (3)	H1A—C12—H1B	109.5
C18—C19—H19	119.4	C5—C12—H1C	109.5
C14—C19—H19	119.4	H1A—C12—H1C	109.5
C3—C4—H4A	109.5	H1B—C12—H1C	109.5
